# Oncogenic seRNA functional activation: a novel mechanism of tumorigenesis

**DOI:** 10.1186/s12943-020-01195-5

**Published:** 2020-04-11

**Authors:** Yuan Tan, Yuejin Li, Faqing Tang

**Affiliations:** Department of Clinical Laboratory and Hunan Key Laboratory of Oncotarget gene, Hunan Cancer Hospital & The affiliated Cancer Hospital of Xiangya School of Medicine, Central South University, Changsha, 410013 China

**Keywords:** Super-enhancer, seRNA, Molecular mechanisms, Cancer progress

## Abstract

seRNA is a noncoding RNA (ncRNA) transcribed from active super-enhancer (SE), through which SE exerts biological functions and participates in various physiological and pathological processes. seRNA recruits cofactor, RNA polymerase II and mediator to constitute and stabilize chromatin loop SE and promoter region, which regulates target genes transcription. In tumorigenesis, DNA insertion, deletion, translocation, focal amplification and carcinogen factor mediate oncogenic SE generation, meanwhile, oncogenic SE transcribes into tumor-related seRNA, termed as oncogenic seRNA. Oncogenic seRNA participates in tumorigenesis through activating various signal-pathways. The recent reports showed that oncogenic seRNA implicates in a widespread range of cytopathological processes in cancer progression including cell proliferation, apoptosis, autophagy, epithelial-mesenchymal transition, extracellular matrix stiffness and angiogenesis. In this article, we comprehensively summarized seRNA’s characteristics and functions, and emphatically introduced inducible formation of oncogenic seRNA and its functional mechanisms. Lastly, some research strategies on oncogenic seRNA were introduced, and the perspectives on cancer therapy that targets oncogenic seRNA were also discussed.

## Background

Typical enhancer is a class of regulatory DNA sequences, its specific functional states are distinguished by a series of histone modifications characteristics [1, 2]. Super enhancer (SE) is enriched with large clusters of enhancers. SE was primarily isolated via the Rank Ordering of SE (ROSE) algorithm in murine embryonic stem cells (ESCs) in 2013 [3, 4]. It is strongly occupied with aberrant high levels of master transcription factors (TFs) (Oct4, Sox2 and Nanog), active histone marks [histone H3 lysine 4 monomethylation (H3K4me1), histone H3 lysine 27 acetylation (H3K27ac)], and transcription regulator factors (cyclin-dependent kinases (CDK)7, Mediator (MED)1, bromodomain-containing protein 4 (BRD4), polymerase II (Pol II) and p300) [5, 6]. Currently, SE identification is mainly dependent on chromatin immunoprecipitation followed by sequence analysis (CHIP-seq) [7, 8].

Classic enhancer not only regulates the transcription of target genes but also actively transcribes into enhancer RNA (eRNA). Consistently, SE also transcribes into ncRNA termed as super enhancer RNA (seRNA) [9], comprising circular RNA (circRNA), long noncoding RNA (lncRNA) and microRNA (miRNA), which play a significant role in gene expression, splicing, translation, and epigenetic regulation [10–12]. Of note, seRNA is characterized by histone modifications (H3K27ac, H3K4me1 and H3K4me2) and chromatin factors [cohesin, p300, CREB-binding protein (CBP) and RNA Pol II] [13, 14]. DNA translocations, small insertions and deletions (indels), focal amplification, single-nucleotide polymorphisms (SNPs), TFs implication and viral infections mediate aberrant SE generation, and the SE further transcribes into seRNA [15–17]. The recent studies have discovered two types of seRNA, *cis*-acting and *trans*-acting seRNA [18]. Meanwhile, according to different transcriptional directions, seRNA is defined as 1d- and 2d-seRNA [19]. Even though, there are some overlapping regions between seRNA and ncRNA, genome-wide sequencing at transcription start site (TSS) loci can distinguish seRNA from ncRNA [20, 21]. Generally, novel technologies to identify seRNA include CHIP-seq [22], CAGE-seq [23], DNase-seq [24], GRO-seq [25], PRO-seq [26], NET-seq [27], mammalian NET-seq (mNET-seq) [28], BruUV-seq [29], and XR-seq [30].

Generally, the distance between seRNA target gene and SE is within 50 kilobase (kb). Nevertheless, there are controversies about target gene position. For one thing, SE may cover TSS of protein-coding gene, for another thing, the regulated genes might be within a segment 50 kb upstream or downstream of the SE [8]. Although actual functional specialization and evolutionary origins of seRNA still remain to be explored, accumulating observations demonstrate that seRNA expression is closely associated with target genes expression via controlling SE activity and facilitating chromatin loop [31, 32]. seRNA plays an essential role in a wide range of physiological and pathological activities. For instance, human SE-lncRNA CARMEN (Cardiac mesoderm enhancer-associated non-coding RNA) participates in cardiac specification, differentiation and homeostasis [33]. In addition, seRNA functions an indispensable role in tumorigenesis through mediating activation of oncogenic signaling pathways, which participates in cell proliferation, autophagy, apoptosis, EMT, ECM remodeling, and angiogenesis. It has been confirmed that seRNA from urothelial cancer associated 1 (UCA1) promotes ovarian cancer development through interacting with angiomotin (AMOT) to activate yes-associated protein (YAP) signaling [34]. To comprehensively clarify the functional mechanisms of seRNA in promoting cancer progression, we systematically introduced seRNA generation and its characteristics, inducible factors of seRNA and their molecular mechanisms in cancer progress. And we also introduced some mysteries to be solved in seRNA research and declared perspectives in cancer therapy targeting oncogenic seRNA.

## seRNA’s characteristics and its functions

Typically, both enhancer and promoter are classified as noncoding elements, yet recent studies indicated that active SE is a novel noncoding element and directionally transcribes into seRNA, respectively [22]. Appreciated with keynote findings, SE is defined based on the high intensity of BRD4, Med1, RNA Pol II, H3K4me1 and H3K27ac [35]. SE transcribes into a group of functional seRNA with different transcriptional modalities, structures and functions, where RNA Pol II mediates the formation of R-loop structure between seRNA and promoter [5]. Notably, some reports demonstrated that production rates of cell type-specific seRNAs mainly depend on enrichment degrees of RNA Pol II [36]. Further, integrator, a multi-subunit complex with a core catalytic RNA endonuclease activity, also plays an indispensable role in biogenesis of mature seRNA and stabilization of SE-promoter chromatin loop via stably combining with C-terminal domain (CTD) of RNA Pol II. GRO-Seq and RNA Pol II profiling showed an accumulated RNA Pol II-seRNA complex and a reduced mature seRNA levels following integrator depletion [37, 38].

Similar to eRNA, seRNA belongs to a class of ncRNA. Nevertheless, there are some similarities and differences between seRNA and ncRNA. Firstly, seRNA is produced by transcription of SE region, displaying a positive correlation of seRNA transcription with histone labeling, especially with H3K27ac modification [39]. Secondly, seRNA and ncRNA have similar transcriptional characteristics at TSS, but seRNA is more unstable and has shorter half-life partly due to RNA exosome activation [40]. Thirdly, ncRNA is predominately spliced and transcribed in one direction. However, seRNA generation is based on unidirectional and bidirectional transcriptions, producing polyadenylated and non-polyadenylated seRNA, respectively [9, 38]. Lastly, SE in transcriptional state enriches transcription initiation complexes and 5-phosphate serine RNA Pol II, which has the characteristics of protein-coding genes promoter [41]. Distinctly, SE-enriched 2-phosphate serine RNA Pol II is less than the whole protein-coding genes. Most importantly, seRNA is labeled with high tissue and cell specificity, it has become one of the most interesting candidates in regulating functional interactions of SE with promoter [42].

**Fig. 1:**
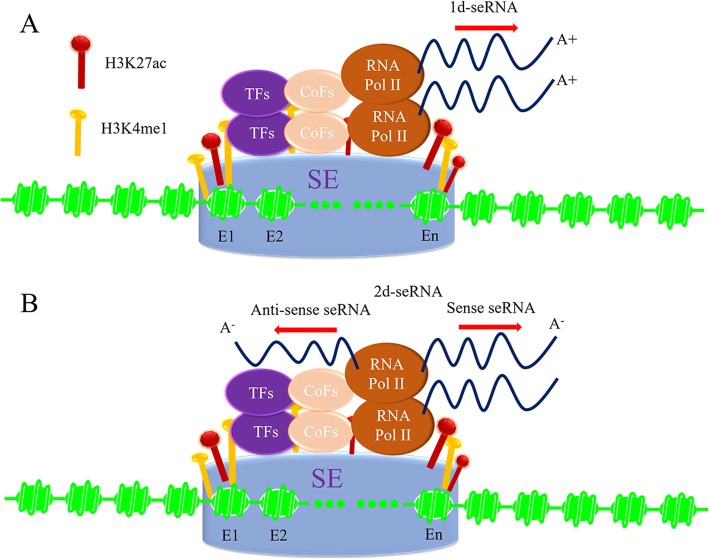
1d-seRNA and 2d-seRNA transcribed from SE regulate gene expression. Active SE enriched with clusters of enhancers absorbs abundant transcription complexes including TFs, CoFs, RNA Pol II, H3K4me1 and H3K27ac modifications. **a**, SE unidirectionally transcribes into 1d-seRNA. **b**, SE induces 2d-seRNA (Anti-sense seRNA and Sense seRNA) transcription

seRNAs mainly contain polyadenylation and non-polyadenylation seRNA (Fig. 1a, b), namely polyA^+^ and polyA^−^ seRNA according to the directions of transcription. PolyA^+^ seRNA is longer than polyA^−^ seRNA, and carries with lower signal ratio of H3K4me1/me3. PolyA^+^ seRNA is unidirectionally transcribed from SE region, also namely 1d-seRNA. While, polyA^−^ seRNA is termed as 2d-seRNA due to bidirectional transcription, it consists of sense and anti-sense seRNA. PolyA^−^ seRNA dose not undergo full maturation and lacks splicing, but it could be modified with 5′ cap [19]. Strikingly, 1d- and 2d-seRNA can simultaneously exist in some diseases, like the existence of p53-regulated 1d- and 2d-seRNA in cancer progress [20].

**Fig. 2:**
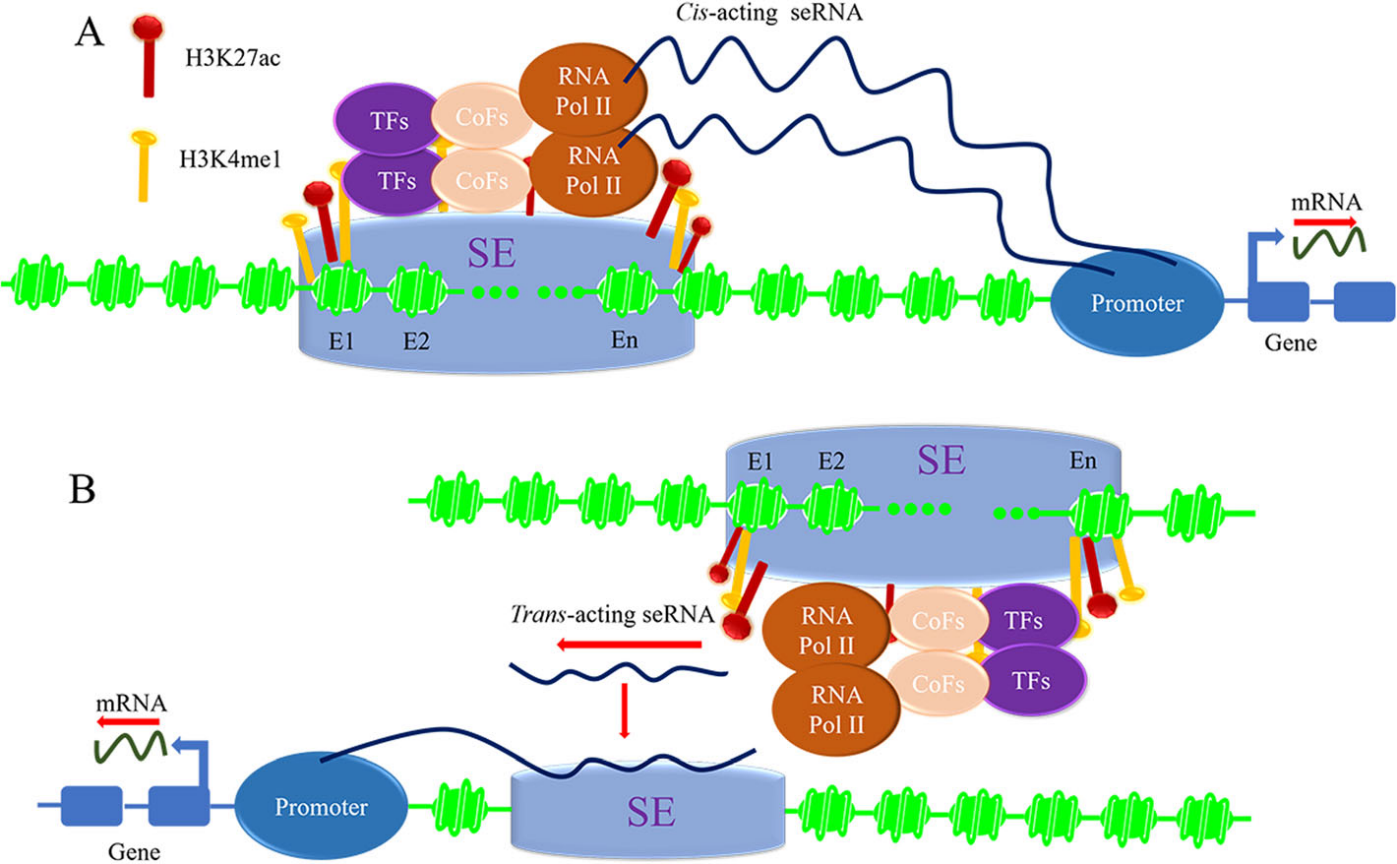
*cis*-acting and *trans*-acting seRNAs transcribed from SE regulate gene expression. Active SE enriches TFs, CoFs, RNA Pol II, H3K4me1 and H3K27ac modifications to regulate gene expression through *cis*-acting and *trans*-acting seRNAs. **a**, *cis*-acting seRNA transcribed from SE regulates adjacent target genes expression. **b**, *trans*-acting seRNA interacts with SE originated from other chromosomes to regulate target genes expression

In addition, seRNAs can be divided into *cis*-acting and *trans*-acting seRNA according to distinct function approaches (Fig. 2a, b) [18]. Cis-acting seRNA recruits protein complexes from its synthetic site to activate adjacent genes, where the whole length or TSS of *cis*-acting seRNA is covered by SE [10]. In embryonic stem cells, non-polyadenylated seRNA produced at SE upstream of Nanog (− 45 enhancer) regulates nearest neighbor *Dppa3* (developmental pluripotency associated 3 gene) via stabilizing the looping of the distal SE at *Dppa3* promoter. Depletion of seRNA reduces *Dppa3* expression [43]. Moreover, a profound study has shown that seRNA could directly interact with CBP in *cis*. The locus-specific binding of CBP with seRNA contributes to the elevated histone acetylation, and directly increases target gene transcription via modulating local chromatin environment [39]. The *trans*-acting seRNA transcribed from local genomic coordinates interacts with SE originated from other chromosomes, which significantly expands functional range of SE [44]. Remarkably, SE-derived polyadenylated alncRNA-EC7/Bloodlinc (seRNA Bloodlinc) amasses at SE to hold *trans* functions, subsequently boosting red blood cell production through binding with heterogeneous nuclear ribonucleoprotein U (HNRNPU) [42]. HNRNP is a nuclear matrix protein that specifically stabilizes seRNA-chromatin associations [42]. Similarly, MYOD Upstream Non-coding RNA (MUNC) is an eRNA transcribed from the upstream of MYOD enhancer. It is observed to induce the expression of specific myogenic genes, like MYOG, and (myosin heavy chain 3) MYH3 that are located on different chromosomes, indicating MUNC acting in *trans* [45]. According to polyadenylated seRNA Bloodlic acting in *trans* and non-polyadenylated seRNA acting in *cis*, there may be a close and complicated correlation between transcriptional directions and function methods of seRNA. Taken together, *cis*-acting seRNA might also exert *trans* functions due to 3D nuclear architecture.

**Fig. 3:**
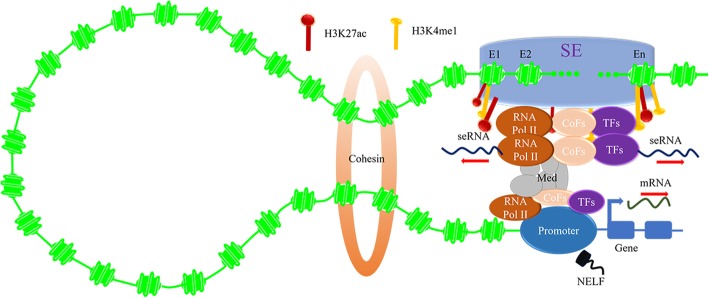
seRNA mediates chromatin loop of SE and promoter. seRNA recruits RNA Pol II, CoFs and MED, forming and stabilizing chromatin loop of SE and promoter. Cohesin complex poises SE and further maintains seRNA-induced loop. seRNA drives out NELF and transiently releases NELF from target genes promoter

seRNA had previously been thought be transcriptional noise that exerts no function due to spurious transcription from open chromatin regions [46]. Currently, it is widely accepted that seRNA exerts a powerful function in forming and stabilizing the chromatin loop, which is confirmed by chromatin conformation capture methods comprising 3C, 4C, 5C and high-throughput chromosome conformation capture (Hi-C) (Fig. 3) [47, 48]. Knockdown of seRNA would disrupt the chromatin loop [5]. Mechanically, SE produces seRNA to bind to promoter, and enhances proximal or distal genes transcription by mediating spatial interaction of SE with promoter in cooperation with RNA Pol II, cofactors (CoFs) and Med [5]. Additionally, accumulating studies have approved that cohesin complex can poise SE, and further maintain seRNA-induced loop [49]. Cohesion knockout would disturb chromosomal loop and target gene activation [50]. Amazingly, seRNA can drive out transcription inhibitory factor negative elongation factor (NELF), and transiently release it from target genes promoter [51]. Clearly, seRNA intimately augments SE function, and appears to be excellent markers of SE activity. In theory, seRNA generation is sensitive to the perturbation of SE, further affecting target genes expression [43].

## Oncogenic seRNA formation

**Fig. 4:**
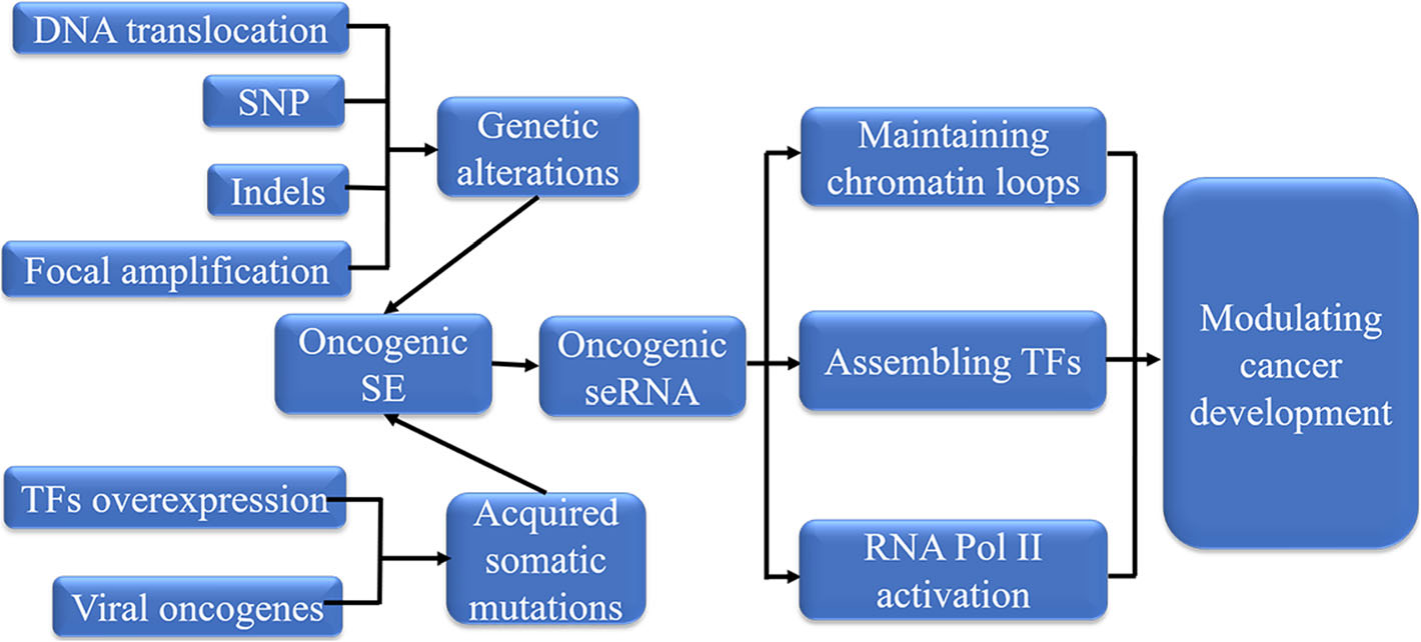
Oncogenic seRNA formation in cancer development. DNA translocation, SNP, Indels, and focal amplification bring out genetic alterations, which mediate oncogenic SE formation and transcribe into oncogenic seRNA. Somatic mutations triggered by viral oncogenes and TFs overexpression generate oncogenic SE to transcribe oncogenic seRNA. Oncogenic seRNAs participate in cancer development via maintaining chromatin loops, assembling TFs and promoting RNA Pol II activation

The aberrant seRNA generated from tumorigenesis, termed as oncogenic seRNA, modulates cancer development via maintaining chromatin loops, assembling TFs and promoting RNA Pol II activation (Fig. 4). Oncogenic seRNA, in one way, is generated from genetic alterations-induced SE, such as SNP, indels, DNA translocation, focal amplification, in other way, it is originated from somatic mutations-generated SE triggered by viral oncogenes and TFs overexpression. SNP is frequently identified within or near SE. SNP rs2168101 resides in SE of the first intron of LIM domain only 1 (LMO1), and SNP rs539846 locates in the intron 3 of B cell lymphoma 2 (BCL2)-modifying factor (BMF) SE, both of them influence neuroblastoma and chronic lymphocytic leukemia (CLL) susceptibility, respectively [52, 53]. Additionally, a single-nucleotide mutation in chromosome 4q32 (4q32A > C) is extremely rare, but this mutation attenuates SE activity and prohibits binding of POU2F1 and Yin-Yang 1 (YY1), which downregulates seRNA and enhances the predisposition of thyroid carcinoma (ATC) [54]. Obviously, SNP-activated SE could transcribe into seRNA to implicate in cancer progression.

In cancers, chromosomal translocations activate SEs to mediate dysregulated-expression of oncogenes. For instance, chromosomal translocation t(3;8)(q27;q24) in diffuse large B cell lymphoma (DLBCL) recruits SE via MYC-BCL6 fusion gene [55], chromosomal translocation t(8;14) in myeloma transfers immunoglobulin H (IgH) SE to breakpoint at 8q24 near MYC loci [56], DNA translocation t(6;8)(p21;q24) in blastic plasmacytoid dendritic cell neoplasm (BPDCN) produces plasmacytoid dendritic cells (pDCs)-specific RUNX2 SE [57]. All of these chromosomal changes upregulate MYC proto-oncogene. Another analysis discovered that SE-induced MYC over-expression is associated with MYC seRNA-mediated R-loop maintenance [5]. In addition, putative SE and seRNA might be obtained from Indels mutations. A novel report demonstrated that the deletions linked with MYC actively generate SE to further augment MYC expression in multiple myeloma (MM) [58], and the existence of MYC seRNA had been approved [5]. In T cell acute lymphoblastic leukemia (T-ALL), short insertion mutations in noncoding intergenic region of TAL1-specific SE produce a de novo myeloblastosis oncogene (MYB) TF binding motif, followed by the recruitment of MYB and H3K27ac-binding CBP, which is important for SE initiation, seRNA transcription, and TAL1 oncogene expression [16]. Notably, focal amplification of enhancer elements frequently occurs in various cancers, which actually accelerates noncoding genes transcription [59]. The two different focal amplifications of SE 3′ to MYC in lung adenocarcinoma and endometrial carcinoma activate and boosts MYC promoter, which depends on lineage-specific chromatin loops and seRNA generation [7]. Additionally, recurrent focal amplification at chromosome 8q24 forms a NOTCH-bound MYC SE and drives MYC transcription, which might involve with MYC seRNA generation [60]. Thereby, focal amplification might participate in cancer development via promoting seRNA-mediated oncogene expression.

Currently, viral infection is identified to be a chief biological pathogenic factor to facilitate oncogenic SE and seRNA generation. Integration of human papillomavirus (HPV) genomes into cellular chromatin is frequent in HPV-associated cancers [61]. Tandemly integrated HPV16 could result in viral-cellular SE element formation [62], which mediates seRNA HOTAIR transcription and enhances E6 and E7 expression, causing cervical cancer pathogenesis [63]. Epstein-Barr virus (EBV) infection promotes EBV-induced SE (ESE) looping, leading to continuous proliferation of lymphoblastoid cell lines (LCLs) [64]. Gro-seq data of LCLs showed that affluent seRNA transcribed at MYC ESE promotes MYC oncogene expression [5]. Interestingly, EBV infection also induces nasopharyngeal carcinoma (NPC)-specific SE generation in ETV6 introns and coding regions, which increases ETV6 expression correlated with poor prognosis [65]. It has well been established that human immunodeficiency virus type 1 (HIV-1) recurrently activates target genes via integrating into proximity of SE in CD4 + T cells [66]. Actually, interferon-regulatory factor 1 (IRF1)/nuclear factor kappa-B (NF-κB) complex at the SE sites is necessary for full HIV-1 SE site-mediated seRNA transcription [67]. Additionally, human lymphotropic virus type I (HTLV-I) is frequently incurable in adult T cell leukemia/lymphoma (ATLL). HBZ and HTLV-I-encoded TFs integrate into ATLL-specific BATF3 SE, further enhancing MYC expression by linking with BATF3/IRF4. Overexpressed MYC exacerbates disease through MYC seRNA transcription [68]. Interestingly, the nuclear matrix protein SAFA (also known as HNRNPU) displays an antiviral function by promoting immunity and stimulating productions of SE and seRNA of antiviral genes, including type I IFNs [69]. Of crucial note, integrating of overexpressed TFs in SE is commonly found in cancer. Particularly, in the patients with B-cell ALL, high ratios of active STAT5 to NF-κB or IKROS in SE also tend to strengthen seRNA expression, and show more aggressive disease phenotypes [70]. NF-κB is a critical TF for driving gene expression, which is involved with SE and seRNA formation [71].

## Functions and mechanisms of oncogenic seRNA in cancer progress

**Fig. 5:**
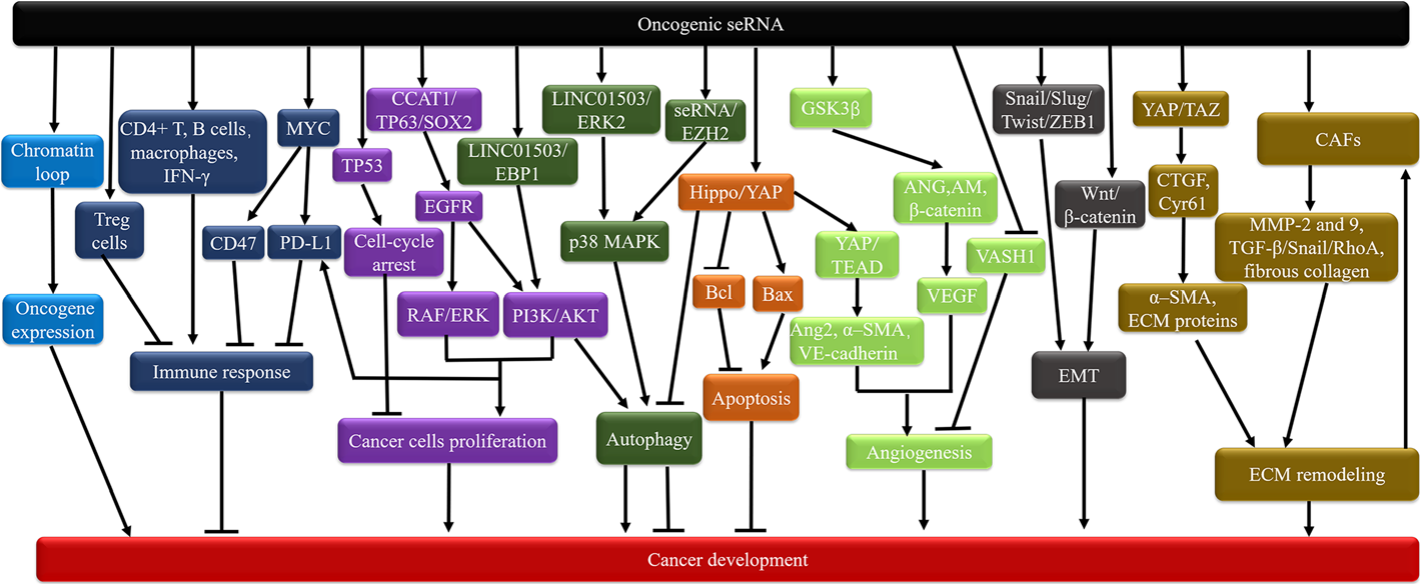
Oncogenic seRNA participates in carcinogenic processes through activating various signal-pathway. Oncogenic seRNA mediates chromatin loops formation to regulate oncogene expression, inducing cancer development. seRNA in Treg cells mediates immunosuppression. seRNAs existing in CD4+ T, B cells and macrophages mediate anticancer immunity through upregulating IFN-γ. seRNA-mediated MYC upregulates CD47 and PD-L1 to inhibit immunity. seRNA from *TP53* SE strengthens *TP53* transcription to induce cell-cycle arrest, consequently suppressing cell proliferation. seRNA CCAT1/TP63/SOX2 complex enhances EGFR transcription and activates RAF/ERK and PI3K/AKT signal pathway, which enhances cancer cells proliferation. seRNA LINC01503/EBP activates PI3K/AKT signaling, seRNA LINC01503/ERK2 and seRNA/EHZ2 activate p38 MAPK signaling, these pathways accelerate autophagy. seRNA-mediated Hippo/YAP induces autophagy inhibition, and regulates apoptosis via Bax and Bcl-2. seRNA-conducted Hippo/YAP also induces angiogenesis via enhancing Ang2, VE-cadherin and α–SMA expression. seRNA downregulates VASH1 to facilitate angiogenesis. SE-mediated GSK-3β drives angiogenesis by triggering ANG, AM, β-catenin pathways and upregulating VEGF. seRNA accelerates EMT by upregulating Snail, Slug, ZEB1 and Twist1 or enhancing Wnt/β-catenin signaling. seRNA-induced YAP/TAZ upregulates CTGF and Cyr61 to promote α–SMA overexpression and ECM protein deposition, accelerating ECM remodeling. seRNA drives CAFs activation to mediate ECM remodeling via MMP-2,9 and TGF-β/Snail/RhoA activation. There is a positive feedback loop between a stiff ECM and CAFs activation

Although the biological function of seRNA still remains poorly characterized, some interesting observations have evidently indicated that seRNA promotes target gene transcription not only to participate in physiological activity, but also to involve in tumorigenic action, including oncogene expression, cancer cell proliferation, EMT, ECM remodeling, angiogenesis, immune response, apoptosis and autophagy (Fig. 5, Table 1).

**Table 1:** The molecule mechanisms of seRNA regulating cancer process

Biological functions	seRNA’s name	Locations	Effects on cancer development	Mechanisms	Ref
EMT	HCCL5	HCC	Exacerbate	Up-regulating Snail, Slug, ZEB1 and Twist1 expression.	[72]
	CCAT1-L	Bladder, cervical and ovarian cancer	Exacerbate	Promoting invasion and metastasis.	[73–75]
	circRNA	HCC	Exacerbate	YY1/p65/p300 complex promotes circRNA transcription.	[76]
Apoptosis	UCA1	NB and GC	Alleviate	Enhancing AMOTp130-YAP and Hippo-YAP activity.	[77, 78]
Autophagy	UCA1	Breast carcinoma	Exacerbate	UCA1-induced Hippo-YAP activity suppresses autophagy.	[79, 80]
	LINC01503	SCC	Exacerbate	Accelerating autophagy via activating PI3K/AKT and ERK/p38 MAPK signaling.	[80, 81]
Angiogenesis	circNfix	ATC and RCC	Exacerbate	Activating GSK3β-mediated β-catenin, ANG and AM signaling and up-regulating VEGF.	[76, 82, 83]
	UCA1	PDAC	Exacerbate	Enhancing Hippo-YAP activity and YAP-TEAD interaction.	[84]
Immune response	seRNA	CD4+ T and Treg cells	Alleviate	Regulating the T and Treg cells differentiation, maturation.	[85, 86]
	seRNA	B cells	Alleviate	Enhancing B cells activation and humoral immunity.	[87–89]
	seRNA	Macrophages	Alleviate	Driving immunity and enhancing the release of IFN-γ.	[90]
	seRNA	IFN-γ	Alleviate	Enhancing function of CD4+ T and NK cells.	[91]
	CCAT1	PD-L1	Exacerbate	Up-regulating PD-L1 by activating PI3K/AKT and RAF/MEK/ERK signaling.	[92]
	CCAT1	PD-L1, CD47	Exacerbate	Up-regulating PD-L1 and CD47 by inducing MYC.	[93]
ECM	UCA1	GC, CRC, lung and breast cancer	Exacerbate	Up-regulating α–SMA and ECM proteins.	[94, 95]
	seRNA	Brest cancers	Exacerbate	Driving CAFs proliferation and myofibroblast differentiation.	[96–98]
Oncogene expression	MYC- seRNA	LCLs	Exacerbate	Promoting transcriptional activation of MYC oncogene.	[5]
	CCAT1-L	CRC	Exacerbate	Assembling CTCF and up-regulating MYC.	[32]
Cancer cells proliferation	CCAT1	SCC	Exacerbate	Forming CCAT1/TP63/SOX2 complex to activate EGFR-induced RAF/MEK/ERK and PI3K/AKT signaling.	[24]
	TP53- seRNA	Various cancers	Alleviate	Increasing TP53 transcription and inducing cell-cycle arrest.	[99]

*HCC* hepatocellular carcinoma, *AMOT* angiomotin, *UCA1* urothelial cancer associated 1, *NB* neuroblastoma, *GC* gastric cancer, *SCC* squamous cell carcinoma, *ATC* anaplastic thyroid carcinoma *RCC*, renal cell carcinoma, *PDAC* pancreatic ductal adenocarcinoma, *Treg* foxp3+ regulatory T, *α–SMA* α-smooth muscle actin, *LCLs* lymphoblastoid cell lines, *EMT* epithelial-mesenchymal transition, *ECM* extracellular matrix, *YAP* yes-associated protein, *TAZ* transcriptional coactivator with PDZ-binding domain, *EGFR* epidermal growth factor receptor, *CAFs* cancer associated fibroblasts, *PI3K* phospholipids inositol triphosphate kinase, *AKT* protein kinase B, *MAPK* mitogen-activated protein kinase, *ERK* extracellular signal regulated kinase, *MEK* mitogen-activated extracellular signal-regulated kinase, *CTCF* CCCTC-binding factor, *VEGF* vascular endothelial growth factor

### seRNA promotes oncogene expression

Oncogenic seRNA functions as a significant regulatory factor for targeting oncogene transcription (Fig. 5). It has been verified that oncogenic EBV infection controls B cells growth and drives lymphoma and carcinoma development via inducing seRNA production and oncogenic MYC expression [64]. Gro-seq data of LCLs revealed that abundant seRNAs transcribed at MYC ESE promote transcriptional activation of MYC oncogene. While knockdown of MYC seRNA significantly attenuates MYC expression via inhibiting MYC ESE looping to MYC TSS [5]. In general, seRNA can recruit TFs to maintain chromatin loops. For instance, colorectal cancer (CRC)-specific seRNA CCAT1-L is classified as a nuclear-retained lncRNA, and 3C analysis showed that CCAT1-L locates at 335 kb upstream of MYC promoter (MYC-335). There is a strongest chromatin interaction between MYC-335 and the MYC promoter, while the interaction between MYC-515 and MYC-355 ranks in the second. Interestingly, CCAT1-L *cis* overexpression remarkably upregulates MYC and accelerates CRC tumorigenesis [32]. Further investigation revealed that CCCTC-binding factor (CTCF) is enriched at the loops of MYC promoter and the MYC-335 and MYC-515 segments, and there is a specific interaction between CTCF and CCAT1-L. CTCF knockdown significantly decreases the transcription of MYC and CCAT1-L. Moreover, depletion of CCAT1-L markedly decreases CTCF occupation of loop regions at MYC. It could be speculated that CCAT1-L may regulate MYC expression by interacting with CTCF, which stabilizes long-range chromatin interactions of MYC promoter with MYC-335 or interaction of MYC-335 with MYC-515 [32]. Additionally, T-ALL-related TAL1 [16], Ewing sarcoma-related MEIS1 [100], hepatocellular carcinoma (HCC)-correlated sphingosine kinase 1 (SPHK1) [101], HPV-induced E6 and E7 [61], oral squamous cell carcinoma (OSCC)-associated PAK4, RUNX1, DNAJB1, SREBF2 and YAP1 [102] are correspondingly regulated by oncogenic SE, and promote cancer development.

### seRNA participates in cancer cell proliferation

Oncogenic seRNA promotes cancer cells proliferation through regulating signal molecules expression and activating signal-pathways (Fig. 5). CCAT1 seRNA is proved to be a significant biomarker in CRC, abundant studies have proved that it is also upregulated in different cancers, such as bladder cancer [73], esophageal cancer [74], cervical cancer [74], prostate cancer [103], and ovarian cancer [75]. In particular, squamous cell carcinoma (SCC) specific SE regions are cooperatively occupied with TP63 and SOX2 to boost CCAT1 seRNA transcription, CCAT1/TP63/SOX2 complex is bound to SE regions of epidermal growth factor receptor (EGFR) to promote EGFR transcription. The overexpressed EGFR contributes to the activation of RAF/mitogen-activated extracellular signal-regulated kinase (MEK)/ERK1/2 and PI3K/AKT signaling pathways, and boosts SCC cell proliferation both in vitro and in vivo [24]. Experimentally, CCAT1 knockdown significantly decreases cell proliferation and colony growth, and reduces volume and mass of the xenografted tumors in vivo, CCAT1 highlights a strong oncogenic potential in SCC cells.

Interestingly, SE regions of several cancer-correlated genes can directly produce seRNA. TIAM2 was identified as an uncharacterized gene in ATL, its overexpression promoted cell proliferation via inducing SE and seRNA activation [104]. CDK inhibitor, THZ1, efficiently downregulates the expression of SE-associated TIAM2 and inhibits cell growth. On the contrary, *TP53*, a tumor suppressor, might produce seRNA from SE regions at p53-dependent manner. The seRNA produced from *TP53* SE regions strengthens efficient *TP53* transcription and induces p53-dependent cell-cycle arrest, showing the potent function of *TP53* SE-transcribed seRNA in suppressing cancer cells proliferation [99]. Collectively, seRNAs transcribed from SE may play a dual role in cancer cells proliferation, but this needs more direct evidence.

### seRNA exerts dual-functions of apoptosis and antiapoptosis

seRNA exerts a apoptosis regulator through modulating several apoptosis mediators such as Bax and Bcl-2 (Fig. 5). seRNA UCA1 highly expresses in various cancers including gastric and ovarian cancer. The direct binding of seRNA UCA1 to AMOT p130 enhances AMOTp130-YAP interaction, which prominently activates Hippo-YAP signaling via promoting YAP dephosphorylation and nuclear translocation [34, 94]. YAP activation significantly upregulates proapoptotic protein Bax expression, downregulates antiapoptotic protein Bcl-2 expression (Fig. 5). The increased Bax/Bcl-2 ratio exerts proapoptosis function in neuroblastoma (NB) and gastric cancer (GC) [77, 78]. Interestingly, activation of mitogen-activated protein kinase (MAPK) signaling inhibits YAP phosphorylation and promotes YAP nuclear translocation via upregulating c-Jun N-terminal kinase (JNK) and extracellular signal regulated kinase (ERK). Hence, the crosstalk between Hippo-YAP and MAPK signaling pathway cooperatively takes part in the regulation of apoptosis behavior in cancer progress [59].

Upon apoptosis stimuli, Bak and Bax form complex, and the accumulation of Bak protein on mitochondrial outer membrane further boosts apoptosis by stimulating the release of proapoptotic proteins from mitochondria into cytosol [105]. To our surprise, SE inhibitors, JQ1 and THZ1, have a potent capability to trigger cancer cells apoptosis accompanied with increased Bax [106], suggesting that SE might block cancer cells apoptosis via upregulating seRNA and proapoptotic protein expression. Thereby, the exact contribution of seRNA to apoptosis might be a “double-edged sword”, and this remains to be explored (Fig. 5).

### seRNA participates in autophagy regulation

Recent studies have found that seRNA expression is tightly associated with autophagy. seRNA UCA1-activated Hippo-YAP is associated with not only apoptosis, but also autophagy. Increased Hippo-YAP activation has been found to control autophagy, which involves in mammalian target of rapamycin (mTOR) pathway that is a notable regulator of autophagy [107]. A study on breast carcinoma MCF-7 cells confirmed that scutellarin treatment upregulates p-YAP and downregulates YAP levels, which represses cancer development via inducing autophagy [79]. Oppositely, UCA1-induced Hippo-YAP activation could suppress autophagy and exacerbate cancer process [80]. SCC-specific seRNA LINC01503 is activated when TF TP63 is bound to SE at seRNA locus, further enhancing malignant phenotype of SCC. Mechanically, overexpressed LINC01503 interacts with ERK2, which leads to activation of ERK/p38 MAPK signaling through inhibiting the binding of ERK2 with dual specificity phosphatase 6 (DUSP6) and reducing ERK2 dephosphorylation (Fig. 5). Similarly, the interaction of LINC01503 with enhancer binding protein (EBP)1 disrupts the binding of EBP1 to p85 subunit of PI3K and promotes PI3K ubiquitination, subsequently activating PI3K/AKT signaling. The two signaling pathways synergistically accelerate autophagy and strengthen oncogenic activity of SCC [80, 81]. In addition, the enhancer of zeste homolog 2 (EZH2) mediates p38 MAPK activation via directly binding with seRNA, and the activated EZH2 induces autophagy through promoting p38 MAPK phosphorylation, following the upregulated autophagy genes including Agt5 and LC-3II [108, 109] (Fig. 5). Disturbance of autophagy-lysosome flux leads to endoplasmic reticulum (ER) stress and an unfolded protein response (UPR), which finally leads to apoptotic cell death in the tumor tissue [110]. In particular, genome stress with temozolomide (TMZ) synergistically induces apoptosis in collaboration with accumulated ER stress with chloroquine treatment [111].

### seRNA mediates EMT of cancer cell

EMT is a reversible trans-differentiation of polarized epithelial cells to mesenchymal cells, which is involved with embryogenesis, wound healing, oncogenes and tumor-suppressor genes expression [112]. Increasing reports indicated that dysregulated seRNA impacts epithelial plasticity by affecting various EMT markers expression (Fig. 5). CRC-specific seRNA CCAT1-L has been proved to be overexpressed in various cancers including bladder, cervical and ovarian cancer, it promotes EMT activation, invasion and metastasis [73–75]. seRNA HCCL5 is considered as an SE-driven cytoplasmic lncRNA in HCC, and it accelerates EMT phenotype, invasion and metastasis in HCC cells by up-regulating Snail, Slug, ZEB1 and Twist1 expression [72]. Interestingly, SE-induced circRNA participates in regulating EMT process. A profound study has discovered that nuclear TF YY1 is bound to SE to build YY1/p65/p300 complex, which facilitates SE-associated circRNA generation to promote the malignancy of HCC [76].

Beyond all doubt, seRNA-correlated oncogenes also exert a positive part in EMT process. CTNND1 (delta-catenin) functions as a novel oncogene in HCC. Notably, knockdown of CTNND1 prominently leads to mesenchymal-epithelial transition (MET), whereas its overexpression enhances EMT and metastatic and invasive properties of HCC via indirectly modulating Wnt/β-catenin signaling, accompanied with increased cyclin D1 and matrix metalloproteinase (MMP)-7 [113, 114]. Previous study has found that canonical Wnt/β-catenin signaling enhances metastasis of cancer cells by up-regulating ZEB1 in vitro [115]. Thus, seRNA may induce CTNND1 further to stimulate Wnt/β-catenin signaling and promote EMT formation through activating ZEB1.

### seRNA regulates cancer angiogenesis

Angiogenesis accelerates cancer progress via providing nutrient and energy supply, thus, it frequently serves as a therapeutic target for cancer [116]. Oncogenic seRNA regulates cancer angiogenesis through activating several signaling pathways (Fig. 5). SE-associated Nfix circRNA (circNfix), namely seRNA Nfix, activates glycogen synthase kinase-3β (GSK-3β) pathway to promote angiogenesis [12, 76]. seRNA-activated PI3K/AKT signaling can not only promote autophagy, but also accelerate angiogenesis in anaplastic ATC and renal cell carcinoma (RCC) through triggering GSK3β/ANG and GSK3β/AM pathway activation [82, 83]. Additionally, GSK3β/β-catenin signaling pathway also enhances angiogenesis through mediating vascular endothelial growth factor (VEGF) expression [117].

In addition, there are other signal pathways that are involved in angiogenesis. seRNA UCA1-activated Hippo-YAP signaling has been proved to induce angiogenesis in pancreatic ductal adenocarcinoma (PDAC) via enhancing Ang2, VE-cadherin and α-smooth muscle actin (α–SMA) expression [84]. seRNA directly binds with EZH2, and the seRNA/EZH2 complex recruits methyl groups to the promoter region of angiogenesis inhibitor gene vasohibin-1 (VASH1), then the reduced VASH1 expression facilitates angiogenesis [118].

### seRNA participates in immune response

Cell specific seRNAs implicate in proliferation, differentiation, maturation and activation of immune cells and secretion of cytokines (Fig. 5). seRNA existed in CD4+ T and foxp3+ regulatory T (Treg) cells plays an important role in T and Treg cells differentiation, maturation and function, respectively [85, 86]. It has been proved that IgH 3΄ regulatory region (3’RR) acts as a major B-cells SE [87], the target genes closer to seRNA are more highly expressed in human humoral immune B cells [88]. Fusion gene ETV6-RUNX1-generated SE induces seRNA generation that is considered as a pivotal marker for CD19^+^/CD20^+^ cells at later stage of B cells differentiation, which is linked with B cells maturation [89]. In macrophages, lipopolysaccharide (LPS)-activated toll-like receptor 4 (TLR4) signaling can facilitate nearly all SE to express seRNA (93.3%) in intergenic regions via recruiting TFs binding, together with overexpression of key genes that drive the releases of innate immunity and inflammatory factor, like IFN-γ [90]. Importantly, IFN-γ seRNA maintains the interaction of NF-κB with IFN-γ locus, which boosts innate and adaptive immune responses against cancer progression [119]. Preclinical data showed that BET inhibitor JQ1 prominently abrogates BRD4-associated IFN-γ seRNA and IFN-γ production via suppressing RNA Pol II binding to the IFN-γ locus, which results in dysfunction of CD4+ T and NK cells, following by the weak immune response [91].

In addition, seRNA manipulates the expression of immune checkpoints, including stimulatory and inhibitory checkpoints [120]. For example, seRNA CCAT1-L-induced MYC upregulates the expression of innate immune checkpoint CD47 (cluster of differentiation 47) and adaptive immune checkpoint PD-L1 (programmed death-ligand 1) by directly interacting with promoters of these two genes in *cis* [93]. Moreover, the CCAT1/TP63/SOX2 complex binds to SE sites of EGFR to enhance EGFR transcription in *trans* [24], further increasing PD-L1 expression by activating PI3K/AKT and RAF/MEK/ERK signaling. Taken together, seRNA CCAT1 could heighten PD-L1 transcription by forming an seRNA-TF complex to promote target genes expression and stimulate downstream signaling pathways [92]. seRNA-associated IFN-γ signaling primarily induces PD-L1 expression in melanoma cells through activating Janus kinase (JAK)-signal transducer and activator of transcription (STAT)-IRF1 axis [121].

It has been demonstrated that BRD is an extremely important constitute of SE, treatment with BRD inhibitor or BRD4 knockdown suppresses PD-L1 expression in ovarian cancer [122]. As being described previously, BRD4 promotes seRNA transcription, and there is a chromatin loop between distal SE and PD-L1 TSS. Therefore, seRNA might be involved in BRD4-mediated PD-L1 up-regulation by maintaining the chromatin loop [123]. Collectively, seRNA suppression mediated by BRD4 inhibitors might promote anticancer immunity by suppressing PD-L1 expression or block anticancer immunity through inactivating immune cells.

### seRNA involves in ECM remodeling

ECM is a crucial component of tumor microenvironment (TME) and an important barrier for invasion and metastasis [124]. seRNA can directly or indirectly influences ECM remodeling via regulating ECM proteins transcription (Fig. 5). Nowadays, several lncRNAs enriched at SE regions have been identified in hepatic stellate cells (HSCs), which are unidirectional seRNAs that encode key genes to regulate ECM stiffness [125]. Currently, a novel study focused on the function of seRNA UCA1-activated YAP, and discovered that aberrant activation of YAP/TAZ (transcriptional coactivator with PDZ-binding domain) axis exists in the microenvironment of various cancers including GC, CRC, lung cancer and breast cancer [94]. YAP/TAZ activation remarkably increases contractile activity and upregulates connective tissue growth factor (CTGF) and Cyr61, which promotes α–SMA overexpression and ECM proteins deposition including laminin, collagen type I and fibronectin [126]. Of critical note, SE-boosted seRNA might drive cancer-associated fibroblasts (CAFs) proliferation and myofibroblast differentiation [96]. This process also accompanies with degradation and remodeling of ECM via secreting MMP-2 and 9 and boosting TGF-β/Snail/RhoA signaling, which accelerates the invasion and metastasis of breast cancer [97, 98]. Amazingly, there is a positive feedback loop between stiff ECM and CAFs activation [95].

As mentioned previously, the pathological role of CAFs in TME was used to consider as a therapeutic strategy for preventing cancer development and progression [127]. Typically, CAFs produce excessive amounts of fibrous collagen, which can be cross-linked by lysyl oxidase (LOX), then increasing focal adhesions and ECM stiffness [128, 129]. In turn, the increased ECM stiffness was identified to profoundly facilitate cancer progression through triggering oncogenic signal pathways including activated focal adhesion kinase (FAK), β-catenin, and PI3K/AKT [129, 130]. Functionally, targeting ECM stiffness via inhibiting LOX enzymatic activity and repressing CAFs proliferation and subsequent CAFs−neoplastic cells interaction, have been demonstrated to decrease metastatic dissemination of breast and colorectal tumor cells in vivo [102, 129].

Of note, PLX4720 (BRAF inhibitor) also leads to activation of CAFs and enhancement of matrix remodeling via negatively affecting BRAF expression. The remodeled matrix enables melanoma cells to tolerate PLX4720 via stimulating integrin β1/FAK-dependent ERK/MAPK signaling [131]. More importantly, the patient-derived tumor xenografts (PDXs) model revealed that co-inhibition of BRAF and FAK abolishes ERK reactivation in tumor stroma [132].

## Challenge and prospective

Recently, seRNA emerges in lots of hot fields due to its wide and strong functions in universal conditions. 3D nuclear architecture studies suggested that seRNA may not only play a role in linear nearby genes expression, but also affect the linear distant genes expression. CRISPR/Cas9 genome-editing technology by disrupting SE functional fragments provides new insights for the exploration of seRNA [133]. In the study on seRNA, several challenges still lie ahead. For instance, transcripts from seRNA are unstable and frequently aborted, which brings immense challenges to find more significant seRNA and validate the corresponding functions [29]. Thereby, future study should focus on postponing seRNA decay, which might involve in RNA metabolism and RNA regulatory pathways [134]. Moreover, it still needs to be verified whether the stability of SE-promoter interaction impacts seRNA stability via regulating the efficiency of recruiting RNA Pol II and other important TFs.

Numerous models have proposed abroad and powerful biological function of seRNA, but the detailed molecular mechanisms of seRNA actually remain to be explored. It is well established that seRNA forms and maintains R-loop to promote adjacent or distant target gene expression. Notably, the maintained presence of chromatin loop between SE and TSS could facilitate transcription initiation. However, it is put forward that seRNA might negatively regulate target genes expression. Since seRNA extensively exerts functions, its transcription might lead to some unknown alterations of physiological activities, this is difficult to be investigated. seRNA is mainly composed of 1d and 2d-seRNA, or *cis*-acting and *trans*-acting seRNA, moreover, abundant polyA^+^ 1d-seRNA accumulated at SE would hold *trans* functions [42]. Maybe, there is profound association between transcriptional direction and functional methods of seRNA. Therefore, distinguished functional mechanisms of seRNA are really worthy of a profound exploration.

In tumorigenesis, DNA damage response (DDR), gene mutations, and genome instability are associated with seRNA formation and alteration [134], which might lead to abnormal genes expression and drive malignant progress of cancer. Theoretically, seRNA has potential to become a better biomarker for diagnosing cancer than frequently used biomarkers such as mRNA, DNA or protein, and it also presents a novel therapy target for cancer due to the high cell specificity [135]. A wide range of preclinical studies suggest that SE inhibitors, such as BRD4 inhibitor JQ1 [136], CDK7 inhibitor THZ1 [137], mediator-associated CDK8 inhibitor cortistatin A [138], CDK12 inhibitor THZ531 [139] and CDK4/6 inhibitor LEE011 [140], have shown dramatic potential for suppressing seRNA transcription and inhibiting cancer growth. As shown in Table 2, combination therapies with SE inhibitors have entered into clinical trials, which provide a deep insight for anticancer therapy. In addition, considering the structural characteristics of SE, future research should pay attention to elucidate the functions of individual components of SE [135].

**Table 2:** Combinational therapies with SE inhibitors in clinical trials

Drug name	Target	Combination	Disease	Status	Phase	NCT number
FT-1101	BET	Azacitidine	AML, MDS or non-hodgkin lymphoma (NHL)	Completed	1	02543879
CPI-0610	BET	Ruxolitinib	Myelofibrosis	Recruiting	2	02158858
BMS-986158	BET	Nivolumab	Advanced tumors	Recruiting	2	T02419417
RO6870810	BET	Daratumumab	Relapsed/refractory multiple myeloma	Active, not Recruiting	1	03068351
SY-1365	CDK7	Carboplatin or Fulvestrant	Advanced solid tumors, ovarian cancer, breast cancer	Recruiting	1	03134638
CT7001	CDK7	Fulvestrant	Advanced solid malignancies	Recruiting	2	03363893
BCD-115	CDK8/19	Endocrine therapy	Breast cancer	Completed	1	03065010
PD-0332991/ Palbociclib	CDK4/6	Binimetinib	Lung cancer	Recruiting	1	03170206
LEE011/Ribociclib	CDK4/6	Ceritinib	Non-small cell lung cancer	Completed	1	02292550
PD-0332991/ Palbociclib	CDK4/6	Nab-Paclitaxel	Metastatic pancreatic ductal adenocarcinoma	Completed	1	02501902
Trilaciclib /G1T28	CDK4/6	Etoposide and Carboplatin	Small cell lung cancer	Completed	1b/2a	02499770

*BET* bromodomain and extra-terminal, *CDK* cyclin-dependent kinases. The data originated from: https://clinicaltrials.gov

## Conclusion

Collectively, seRNA derived from active SE has a powerful transcriptional regulation function, and its production rate is based on the recruitment of RNA Pol II. Significantly, seRNA regulates near gene transcription and mediates distant gene expression via forming and maintaining the chromatin loop of SE and promoter. During tumorigenesis, DNA insertion, deletion, translocation, focal amplification and carcinogen factor mediate oncogenic SE generation, and oncogenic SE transcribes into oncogenic seRNA. Oncogenic seRNA activates multiple signaling pathways that are associated with cell proliferation, EMT, apoptosis, autophagy, ECM remodeling, angiogenesis, and immune response, promoting carcinogenesis. SE inhibitors are capable of blocking seRNA generation via disrupting SE to suppress oncogenic signaling pathways, therefore, targeting seRNA might represent new strategies for cancer therapy.

## Data Availability

All data generated or analysed during this study are included in this published article and its supplementary information files.

## Abbreviations

AMOT: Angiomotin
α–SMA: α-smooth muscle actin
ATC: Anaplastic thyroid carcinoma
ATLL: Adult T cell leukemia/lymphoma
BET: Bromodomain and extra-terminal
BMF: B cell lymphoma 2 (BCL2)-modifying factor
BPDCN: Blastic plasmacytoid dendritic cell neoplasm
BRD4: Bromodomain-containing protein 4
BruUV-seq: Bromouridine ultraviolet sequencing
CAFs: Cancer associated fibroblasts
CAGE-seq: Cap analysis of gene expression sequencing
CBP: CREB-binding protein
CCAT1-L: Colon cancer associated transcript 1
CD47: Cluster of differentiation 47
CHIP-seq: Chromatin immunoprecipitation followed by sequence analysis
circNfix: Nfix circRNA
circRNA: Circular RNA
CLL: Chronic lymphocytic leukemia
CoFs: Cofactors
CQ: Chloroquine
CRC: Colorectal cancer
CTCF: CCCTC-binding factor
CTD: C-terminal domain
DLBCL: Diffuse large B cell lymphoma
DNase-seq: DNase I hypersensitive sites sequencing
DUSP6: Dual specificity phosphatase 6
EBP: Enhancer binding protein
EBV: Epstein–Barr virus
ECM: Extracellular matrix
EMT: Epithelial-mesenchymal transition
eRNA: Enhancer RNA
ESCs: Embryonic stem cells
EZH2: Enhancer of zeste homolog 2
FAK: Focal adhesion kinase
GC: Gastric cancer
GRO-seq: Global nuclear run-on sequencing
H3K4me1: Histone H3 lysine 4 monomethylation
H3K27ac: Histone H3 lysine 27 acetylation
HCC: Hepatocellular carcinoma
Hi-C: High-throughput chromosome conformation capture
HNRNPU: Heterogeneous nuclear ribonucleoprotein U
HSCs: Hepatic stellate cells
HTLV-I: Human lymphotropic virus type I
Indels: Insertions and deletions
IRF1: Interferon-regulatory factor 1
JAK: Janus kinase
LCLs: Lymphoblastoid cell lines
lncRNA: Long noncoding RNA
LOX: Lysyl oxidase
LPS: Lipopolysaccharide
MED: Mediator
MEK: Mitogen-activated extracellular signal-regulated kinase
MET: Mesenchymal-epithelial transition
MYB: Myeloblastosis oncogene
MYH3: Myosin heavy chain 3
NB: Neuroblastoma
ncRNA: Noncoding RNA
NELF: Negative elongation factor
NET-seq: Native elongating transcript sequencing
NPC: Nasopharyngeal carcinoma
PARP: Poly ADP-ribose polymerase
PDAC: Pancreatic ductal adenocarcinoma
pDCs: Plasmacytoid dendritic cells
PD-L1: Programmed death-ligand 1
PDXs: Patient-derived tumor xenografts
Pol II: Polymerase II
PRO-seq: Precision nuclear run-on sequencing
RCC: Renal cell carcinoma
ROSE: Rank ordering of SE
SCC: Squamous cell carcinoma
SE: Super-enhancer
seRNA: Super enhancer RNA
SPHK1: Sphingosine kinase 1
STAT: Signal transducer and activator of transcription
TAZ: Transcriptional coactivator with PDZ-binding domain
TFs: Transcription factors
TLR4: Toll-like receptor 4
TME: Tumor microenvironment
TMZ: Temozolomide
TSS: Transcription start site
Treg: Foxp3+ regulatory T
UCA1: Urothelial cancer associated 1
VASH1: Vasohibin-1
XR-seq: Excision repair sequencing
YAP: Yes-associated protein
YY1: Yin-Yang 1

## References

[1] Luo Z, Lin C (2016). Enhancer, epigenetics, and human disease. Curr Opin Genet Dev.

[2] Calo E, Wysocka J (2013). Modification of enhancer chromatin: what, how, and why?. Mol Cell.

[3] Hnisz D, Abraham BJ, Lee TI, Lau A, Saint-Andre V, Sigova AA, Hoke HA, Young RA (2013). Super-enhancers in the control of cell identity and disease. Cell.

[4] Whyte WA, Orlando DA, Hnisz D, Abraham BJ, Lin CY, Kagey MH, Rahl PB, Lee TI, Young RA (2013). Master transcription factors and mediator establish super-enhancers at key cell identity genes. Cell.

[5] Yang Y, Su Z, Song X, Liang B, Zeng F, Chang X, Huang D (2016). Enhancer RNA-driven looping enhances the transcription of the long noncoding RNA DHRS4-AS1, a controller of the DHRS4 gene cluster. Sci Rep.

[6] Peng Y, Zhang Y (2018). Enhancer and super-enhancer: positive regulators in gene transcription. Animal Model Exp Med.

[7] Zhang X, Choi PS, Francis JM, Imielinski M, Watanabe H, Cherniack AD, Meyerson M (2016). Identification of focally amplified lineage-specific super-enhancers in human epithelial cancers. Nat Genet.

[8] Niederriter AR, Varshney A, Parker SC, Martin DM (2015). Super enhancers in cancers, complex disease, and developmental disorders. Genes (Basel).

[9] Mao R, Wu Y, Ming Y, Xu Y, Wang S, Chen X, Wang X, Fan Y (2019). Enhancer RNAs: a missing regulatory layer in gene transcription. Sci China Life Sci.

[10] Soibam B (2017). Super-lncRNAs: identification of lncRNAs that target super-enhancers via RNA:DNA:DNA triplex formation. RNA.

[11] Suzuki HI, Young RA, Sharp PA (2017). Super-enhancer-mediated RNA processing revealed by integrative MicroRNA network analysis. Cell.

[12] Huang S, Li X, Zheng H, Si X, Li B, Wei G, Li C, Chen Y, Chen Y, Liao W (2019). Loss of super-enhancer-regulated circRNA Nfix induces cardiac regeneration after myocardial infarction in adult mice. Circulation.

[13] Ounzain S, Pezzuto I, Micheletti R, Burdet F, Sheta R, Nemir M, Gonzales C, Sarre A, Alexanian M, Blow MJ (2014). Functional importance of cardiac enhancer-associated noncoding RNAs in heart development and disease. J Mol Cell Cardiol.

[14] Rothschild G, Basu U (2017). Lingering questions about enhancer RNA and enhancer transcription-coupled genomic instability. Trends Genet.

[15] Cong Z, Li Q, Yang Y, Guo X, Cui L, You T (2019). The SNP of rs6854845 suppresses transcription via the DNA looping structure alteration of super-enhancer in colon cells. Biochem Biophys Res Commun.

[16] Mansour MR, Abraham BJ, Anders L, Berezovskaya A, Gutierrez A, Durbin AD, Etchin J, Lawton L, Sallan SE, Silverman LB (2014). Oncogene regulation. An oncogenic super-enhancer formed through somatic mutation of a noncoding intergenic element. Science.

[17] Chakravorty S, Yan B, Wang C, Wang L, Quaid JT, Lin CF, Briggs SD, Majumder J, Canaria DA, Chauss D, et al. Integrated pan-cancer map of EBV-associated neoplasms reveals functional host-virus interactions. Cancer Res. 2019;79:6010–23. 10.1158/0008-5472.CAN-19-0615 PMC6891172 31481499

[18] Guo ZW, Xie C, Li K, Zhai XM, Cai GX, Yang XX, Wu YS. SELER: a database of super-enhancer-associated lncRNA- directed transcriptional regulation in human cancers. Database (Oxford). 2019;1:2019. 10.1093/database/baz027 PMC6390648 30806704

[19] Natoli G, Andrau JC (2012). Noncoding transcription at enhancers: general principles and functional models. Annu Rev Genet.

[20] Leveille N, Melo CA, Rooijers K, Diaz-Lagares A, Melo SA, Korkmaz G, Lopes R, Moqadam FA, Maia AR, Wijchers PJ (2015). Genome-wide profiling of p53-regulated enhancer RNAs uncovers a subset of enhancers controlled by a lncRNA. Nat Commun.

[21] Blinka S, Reimer MH, Pulakanti K, Pinello L, Yuan GC, Rao S (2017). Identification of transcribed enhancers by genome-wide chromatin Immunoprecipitation sequencing. Methods Mol Biol.

[22] Le Gras S, Keime C, Anthony A, Lotz C, De Longprez L, Brouillet E, Cassel JC, Boutillier AL, Merienne K (2017). Altered enhancer transcription underlies Huntington's disease striatal transcriptional signature. Sci Rep.

[23] Djavadian R, Hayes M, Johannsen E (2018). CAGE-seq analysis of Epstein-Barr virus lytic gene transcription: 3 kinetic classes from 2 mechanisms. PLoS Pathog.

[24] Jiang Y, Jiang YY, Xie JJ, Mayakonda A, Hazawa M, Chen L, Xiao JF, Li CQ, Huang ML, Ding LW (2018). Co-activation of super-enhancer-driven CCAT1 by TP63 and SOX2 promotes squamous cancer progression. Nat Commun.

[25] Chae M, Danko CG, Kraus WL (2015). groHMM: a computational tool for identifying unannotated and cell type-specific transcription units from global run-on sequencing data. BMC Bioinformatics.

[26] Zhao Y, Liu Q, Acharya P, Stengel KR, Sheng Q, Zhou X, Kwak H, Fischer MA, Bradner JE, Strickland SA (2016). High-resolution mapping of RNA polymerases identifies mechanisms of sensitivity and resistance to BET inhibitors in t(8;21) AML. Cell Rep.

[27] Mylonas C, Tessarz P (2019). NET-prism enables RNA polymerase-dedicated transcriptional interrogation at nucleotide resolution. RNA Biol.

[28] Szlachta K, Thys RG, Atkin ND, Pierce LCT, Bekiranov S, Wang YH (2018). Alternative DNA secondary structure formation affects RNA polymerase II promoter-proximal pausing in human. Genome Biol.

[29] Magnuson B, Veloso A, Kirkconnell KS, de Andrade Lima LC, Paulsen MT, Ljungman EA, Bedi K, Prasad J, Wilson TE, Ljungman M (2015). Identifying transcription start sites and active enhancer elements using BruUV-seq. Sci Rep.

[30] Hu J, Adar S, Selby CP, Lieb JD, Sancar A (2015). Genome-wide analysis of human global and transcription-coupled excision repair of UV damage at single-nucleotide resolution. Genes Dev.

[31] Ounzain S, Pedrazzini T (1863). Super-enhancer lncs to cardiovascular development and disease. Biochim Biophys Acta.

[32] Xiang JF, Yin QF, Chen T, Zhang Y, Zhang XO, Wu Z, Zhang S, Wang HB, Ge J, Lu X (2014). Human colorectal cancer-specific CCAT1-L lncRNA regulates long-range chromatin interactions at the MYC locus. Cell Res.

[33] Ounzain S, Micheletti R, Arnan C, Plaisance I, Cecchi D, Schroen B, Reverter F, Alexanian M, Gonzales C, Ng SY (2015). CARMEN, a human super enhancer-associated long noncoding RNA controlling cardiac specification, differentiation and homeostasis. J Mol Cell Cardiol.

[34] Lin X, Spindler TJ, de Souza Fonseca MA, Corona RI, Seo JH, Dezem FS, Li L, Lee JM, Long HW, Sellers TA (2019). Super-Enhancer-Associated LncRNA UCA1 Interacts Directly with AMOT to Activate YAP Target Genes in Epithelial Ovarian Cancer. iScience.

[35] Huang PP, Brusman LE, Iyer AK, Webster NJ, Mellon PL (2016). A novel gonadotropin-releasing hormone 1 (Gnrh1) enhancer-derived noncoding RNA regulates Gnrh1 gene expression in GnRH neuronal cell models. PLoS One.

[36] Pulakanti K, Pinello L, Stelloh C, Blinka S, Allred J, Milanovich S, Kiblawi S, Peterson J, Wang A, Yuan GC, Rao S (2013). Enhancer transcribed RNAs arise from hypomethylated, Tet-occupied genomic regions. Epigenetics.

[37] Stadelmayer B, Micas G, Gamot A, Martin P, Malirat N, Koval S, Raffel R, Sobhian B, Severac D, Rialle S (2014). Integrator complex regulates NELF-mediated RNA polymerase II pause/release and processivity at coding genes. Nat Commun.

[38] Lai F, Gardini A, Zhang A, Shiekhattar R (2015). Integrator mediates the biogenesis of enhancer RNAs. Nature.

[39] Bose DA, Donahue G, Reinberg D, Shiekhattar R, Bonasio R, Berger SL (2017). RNA binding to CBP stimulates histone acetylation and transcription. Cell.

[40] Belair C, Sim S, Kim KY, Tanaka Y, Park IH, Wolin SL (2019). The RNA exosome nuclease complex regulates human embryonic stem cell differentiation. J Cell Biol.

[41] Lynch CJ, Bernad R, Calvo I, Nobrega-Pereira S, Ruiz S, Ibarz N, Martinez-Val A, Grana-Castro O, Gomez-Lopez G, Andres-Leon E (2018). The RNA polymerase II factor RPAP1 is critical for mediator-driven transcription and cell identity. Cell Rep.

[42] Alvarez-Dominguez JR, Knoll M, Gromatzky AA, Lodish HF (2017). The super-enhancer-derived alncRNA-EC7/Bloodlinc potentiates red blood cell development in trans. Cell Rep.

[43] Blinka S, Reimer MH, Pulakanti K, Rao S (2016). Super-enhancers at the Nanog locus differentially regulate neighboring Pluripotency-associated genes. Cell Rep.

[44] Su ZD, Huang Y, Zhang ZY, Zhao YW, Wang D, Chen W, Chou KC, Lin H (2018). iLoc-lncRNA: predict the subcellular location of lncRNAs by incorporating octamer composition into general PseKNC. Bioinformatics.

[45] Cichewicz MA, Kiran M, Przanowska RK, Sobierajska E, Shibata Y, Dutta A. MUNC, an enhancer RNA upstream from the MYOD gene, induces a subgroup of myogenic transcripts in trans independently of MyoD. Mol Cell Biol. 2018;38:e00655–17. 10.1128/MCB.00655-17 PMC6168980 30037979

[46] Struhl K (2007). Transcriptional noise and the fidelity of initiation by RNA polymerase II. Nat Struct Mol Biol.

[47] Zhang Z, Lee JH, Ruan H, Ye Y, Krakowiak J, Hu Q, Xiang Y, Gong J, Zhou B, Wang L (2019). Transcriptional landscape and clinical utility of enhancer RNAs for eRNA-targeted therapy in cancer. Nat Commun.

[48] Buffry AD, Mendes CC, McGregor AP (2016). The functionality and evolution of eukaryotic transcriptional enhancers. Adv Genet.

[49] Fan J, Xu Y, Wen X, Ge S, Jia R, Zhang H, Fan XA. Cohesin-mediated Intrachromosomal loop drives oncogenic ROR lncRNA to accelerate tumorigenesis. Mol Ther. 2019;27:2182–94. 10.1016/j.ymthe.2019.07.020 PMC6904803 31451355

[50] Li W, Notani D, Ma Q, Tanasa B, Nunez E, Chen AY, Merkurjev D, Zhang J, Ohgi K, Song X (2013). Functional roles of enhancer RNAs for oestrogen-dependent transcriptional activation. Nature.

[51] Leveille N, Melo CA, Agami R (2015). Enhancer-associated RNAs as therapeutic targets. Expert Opin Biol Ther.

[52] Oldridge DA, Wood AC, Weichert-Leahey N, Crimmins I, Sussman R, Winter C, McDaniel LD, Diamond M, Hart LS, Zhu S (2015). Genetic predisposition to neuroblastoma mediated by a LMO1 super-enhancer polymorphism. Nature.

[53] Kandaswamy R, Sava GP, Speedy HE, Bea S, Martin-Subero JI, Studd JB, Migliorini G, Law PJ, Puente XS, Martin-Garcia D (2016). Genetic predisposition to chronic lymphocytic leukemia is mediated by a BMF super-enhancer polymorphism. Cell Rep.

[54] He H, Li W, Wu D, Nagy R, Liyanarachchi S, Akagi K, Jendrzejewski J, Jiao H, Hoag K, Wen B (2013). Ultra-rare mutation in long-range enhancer predisposes to thyroid carcinoma with high penetrance. PLoS One.

[55] Kleinstern G, Yan H, Hildebrandt MAT, Vijai J, Berndt SI, Ghesquieres H, McKay J, Wang SS, Nieters A, Ye Y, et al. Inherited variants at 3q13.33 and 3p24.1 are associated with risk of diffuse large B-cell lymphoma and implicate immune pathways. Hum Mol Genet. 2020;29:70–9. 10.1093/hmg/ddz228 PMC7001601 31600786

[56] Walker BA, Wardell CP, Brioli A, Boyle E, Kaiser MF, Begum DB, Dahir NB, Johnson DC, Ross FM, Davies FE, Morgan GJ (2014). Translocations at 8q24 juxtapose MYC with genes that harbor superenhancers resulting in overexpression and poor prognosis in myeloma patients. Blood Cancer J.

[57] Kubota S, Tokunaga K, Umezu T, Yokomizo-Nakano T, Sun Y, Oshima M, Tan KT, Yang H, Kanai A, Iwanaga E (2019). Lineage-specific RUNX2 super-enhancer activates MYC and promotes the development of blastic plasmacytoid dendritic cell neoplasm. Nat Commun.

[58] Affer M, Chesi M, Chen WG, Keats JJ, Demchenko YN, Roschke AV, Van Wier S, Fonseca R, Bergsagel PL, Kuehl WM (2014). Promiscuous MYC locus rearrangements hijack enhancers but mostly super-enhancers to dysregulate MYC expression in multiple myeloma. Leukemia.

[59] Zhu WQ, Yu YJ, Xu LN, Ming PP, Shao SY, Qiu J (2019). Regulation of osteoblast behaviors via cross-talk between Hippo/YAP and MAPK signaling pathway under fluoride exposure. J Mol Med (Berl).

[60] Chiang MY, Wang Q, Gormley AC, Stein SJ, Xu L, Shestova O, Aster JC, Pear WS (2016). High selective pressure for Notch1 mutations that induce Myc in T-cell acute lymphoblastic leukemia. Blood.

[61] Warburton A, Redmond CJ, Dooley KE, Fu H, Gillison ML, Akagi K, Symer DE, Aladjem MI, McBride AA (2018). HPV integration hijacks and multimerizes a cellular enhancer to generate a viral-cellular super-enhancer that drives high viral oncogene expression. PLoS Genet.

[62] Dooley KE, Warburton A, McBride AA. Tandemly integrated HPV16 can form a Brd4-dependent super-enhancer-like element that drives transcription of viral oncogenes. mBio. 2016;7. 10.1128/mBio.01446-16 PMC5021809 27624132

[63] Sharma S, Mandal P, Sadhukhan T, Roy Chowdhury R, Ranjan Mondal N, Chakravarty B, Chatterjee T, Roy S, Sengupta S (2015). Bridging links between Long noncoding RNA HOTAIR and HPV Oncoprotein E7 in cervical Cancer pathogenesis. Sci Rep.

[64] Jiang S, Zhou H, Liang J, Gerdt C, Wang C, Ke L, Schmidt SCS, Narita Y, Ma Y, Wang S (2017). The Epstein-Barr virus Regulome in Lymphoblastoid cells. Cell Host Microbe.

[65] Ke L, Zhou H, Wang C, Xiong G, Xiang Y, Ling Y, Khabir A, Tsao GS, Zeng Y, Zeng M (2017). Nasopharyngeal carcinoma super-enhancer-driven ETV6 correlates with prognosis. Proc Natl Acad Sci U S A.

[66] Lucic B, Chen HC, Kuzman M, Zorita E, Wegner J, Minneker V, Wang W, Fronza R, Laufs S, Schmidt M (2019). Spatially clustered loci with multiple enhancers are frequent targets of HIV-1 integration. Nat Commun.

[67] Sgarbanti M, Remoli AL, Marsili G, Ridolfi B, Borsetti A, Perrotti E, Orsatti R, Ilari R, Sernicola L, Stellacci E (2008). IRF-1 is required for full NF-kappaB transcriptional activity at the human immunodeficiency virus type 1 long terminal repeat enhancer. J Virol.

[68] Nakagawa M, Shaffer AL, Ceribelli M, Zhang M, Wright G, Huang DW, Xiao W, Powell J, Petrus MN, Yang Y (2018). Targeting the HTLV-I-regulated BATF3/IRF4 transcriptional network in adult T cell leukemia/lymphoma. Cancer Cell.

[69] Cao L, Liu S, Li Y, Yang G, Luo Y, Li S, Du H, Zhao Y, Wang D, Chen J (2019). The nuclear matrix protein SAFA Surveils viral RNA and facilitates immunity by activating antiviral enhancers and super-enhancers. Cell Host Microbe.

[70] Katerndahl CDS, Heltemes-Harris LM, Willette MJL, Henzler CM, Frietze S, Yang R, Schjerven H, Silverstein KAT, Ramsey LB, Hubbard G (2017). Antagonism of B cell enhancer networks by STAT5 drives leukemia and poor patient survival. Nat Immunol.

[71] Brown JD, Lin CY, Duan Q, Griffin G, Federation A, Paranal RM, Bair S, Newton G, Lichtman A, Kung A (2014). NF-kappaB directs dynamic super enhancer formation in inflammation and atherogenesis. Mol Cell.

[72] Peng L, Jiang B, Yuan X, Qiu Y, Peng J, Huang Y, Zhang C, Zhang Y, Lin Z, Li J (2019). Super-enhancer-associated Long noncoding RNA HCCL5 is activated by ZEB1 and promotes the malignancy of hepatocellular carcinoma. Cancer Res.

[73] Hu M, Zhang Q, Tian XH, Wang JL, Niu YX, Li G. lncRNA CCAT1 is a biomarker for the proliferation and drug resistance of esophageal cancer via the miR-143/PLK1/BUBR1 axis. Mol Carcinog. 2019;58:2207–17. 10.1002/mc.23109 31544294

[74] Shen H, Wang L, Xiong J, Ren C, Gao C, Ding W, Zhu D, Ma D, Wang H (2019). Long non-coding RNA CCAT1 promotes cervical cancer cell proliferation and invasion by regulating the miR-181a-5p/MMP14 axis. Cell Cycle.

[75] Lai XJ, Cheng HF (2018). LncRNA colon cancer-associated transcript 1 (CCAT1) promotes proliferation and metastasis of ovarian cancer via miR-1290. Eur Rev Med Pharmacol Sci.

[76] Liu J, Zhao K, Huang N, Zhang N (2019). Circular RNAs and human glioma. Cancer Biol Med.

[77] Ye C, Wang W, Xia G, Yu C, Yi Y, Hua C, Tu F, Shen L, Chen C, Sun W, Zheng Z (2019). A novel curcumin derivative CL-6 exerts antitumor effect in human gastric cancer cells by inducing apoptosis through Hippo-YAP signaling pathway. Onco Targets Ther.

[78] Zhao Q, Jia X, Zhang Y, Dong Y, Lei Y, Tan X, Williamson RA, Wang A, Zhang D, Ma J (2019). Tetrandrine induces apoptosis in human neuroblastoma through regulating the Hippo/YAP signaling pathway. Biochem Biophys Res Commun.

[79] Hou L, Chen L, Fang L (2017). Scutellarin inhibits proliferation, invasion, and Tumorigenicity in human breast Cancer cells by regulating HIPPO-YAP signaling pathway. Med Sci Monit.

[80] Xie JJ, Jiang YY, Jiang Y, Li CQ, Lim MC, An O, Mayakonda A, Ding LW, Long L, Sun C (2018). Super-enhancer-driven Long non-coding RNA LINC01503, regulated by TP63, is over-expressed and oncogenic in squamous cell carcinoma. Gastroenterology.

[81] Kim KY, Park KI, Kim SH, Yu SN, Park SG, Kim YW, Seo YK, Ma JY, Ahn SC. Inhibition of autophagy promotes Salinomycin-induced apoptosis via reactive oxygen species-mediated PI3K/AKT/mTOR and ERK/p38 MAPK-dependent signaling in human prostate Cancer cells. Int J Mol Sci. 2017;18. 10.3390/ijms18051088 PMC5454997 28524116

[82] Chen Y, Li C, Xie H, Fan Y, Yang Z, Ma J, He D, Li L (2017). Infiltrating mast cells promote renal cell carcinoma angiogenesis by modulating PI3K-->AKT-->GSK3beta-->AM signaling. Oncogene.

[83] Jin Z, Cheng X, Feng H, Kuang J, Yang W, Peng C, Shen B, Qiu W (2017). Apatinib inhibits angiogenesis via suppressing Akt/GSK3beta/ANG signaling pathway in anaplastic thyroid Cancer. Cell Physiol Biochem.

[84] Wei H, Wang F, Wang Y, Li T, Xiu P, Zhong J, Sun X, Li J (2017). Verteporfin suppresses cell survival, angiogenesis and vasculogenic mimicry of pancreatic ductal adenocarcinoma via disrupting the YAP-TEAD complex. Cancer Sci.

[85] Witte S, O'Shea JJ, Vahedi G (2015). Super-enhancers: Asset management in immune cell genomes. Trends Immunol.

[86] Kitagawa Y, Ohkura N, Kidani Y, Vandenbon A, Hirota K, Kawakami R, Yasuda K, Motooka D, Nakamura S, Kondo M (2017). Guidance of regulatory T cell development by Satb1-dependent super-enhancer establishment. Nat Immunol.

[87] Le Noir S, Boyer F, Lecardeur S, Brousse M, Oruc Z, Cook-Moreau J, Denizot Y, Cogne M (2017). Functional anatomy of the immunoglobulin heavy chain 3 super-enhancer needs not only core enhancer elements but also their unique DNA context. Nucleic Acids Res.

[88] Agirre X, Meydan C, Jiang Y, Garate L, Doane AS, Li Z, Verma A, Paiva B, Martin-Subero JI, Elemento O (2019). Long non-coding RNAs discriminate the stages and gene regulatory states of human humoral immune response. Nat Commun.

[89] Teppo S, Laukkanen S, Liuksiala T, Nordlund J, Oittinen M, Teittinen K, Gronroos T, St-Onge P, Sinnett D, Syvanen AC (2016). Genome-wide repression of eRNA and target gene loci by the ETV6-RUNX1 fusion in acute leukemia. Genome Res.

[90] Hah N, Benner C, Chong LW, Yu RT, Downes M, Evans RM (2015). Inflammation-sensitive super enhancers form domains of coordinately regulated enhancer RNAs. Proc Natl Acad Sci U S A.

[91] Gibbons HR, Mi DJ, Farley VM, Esmond T, Kaood MB, Aune TM (2019). Bromodomain inhibitor JQ1 reversibly blocks IFN-gamma production. Sci Rep.

[92] Ota K, Azuma K, Kawahara A, Hattori S, Iwama E, Tanizaki J, Harada T, Matsumoto K, Takayama K, Takamori S (2015). Induction of PD-L1 expression by the EML4-ALK Oncoprotein and downstream signaling pathways in non-small cell lung Cancer. Clin Cancer Res.

[93] Casey SC, Tong L, Li Y, Do R, Walz S, Fitzgerald KN, Gouw AM, Baylot V, Gutgemann I, Eilers M, Felsher DW (2016). MYC regulates the antitumor immune response through CD47 and PD-L1. Science.

[94] Yao F, Wang Q, Wu Q (2019). The prognostic value and mechanisms of lncRNA UCA1 in human cancer. Cancer Manag Res.

[95] Noguchi S, Saito A, Nagase T. YAP/TAZ signaling as a molecular link between fibrosis and Cancer. Int J Mol Sci. 2018;19. 10.3390/ijms19113674 PMC6274979 30463366

[96] Felisbino MB, McKinsey TA (2018). Epigenetics in cardiac fibrosis: emphasis on inflammation and fibroblast activation. JACC Basic Transl Sci.

[97] Stanisavljevic J, Loubat-Casanovas J, Herrera M, Luque T, Pena R, Lluch A, Albanell J, Bonilla F, Rovira A, Pena C (2015). Snail1-expressing fibroblasts in the tumor microenvironment display mechanical properties that support metastasis. Cancer Res.

[98] Fan SH, Wang YY, Lu J, Zheng YL, Wu DM, Zhang ZF, Shan Q, Hu B, Li MQ, Cheng W (2015). CERS2 suppresses tumor cell invasion and is associated with decreased V-ATPase and MMP-2/MMP-9 activities in breast cancer. J Cell Biochem.

[99] Melo CA, Drost J, Wijchers PJ, van de Werken H, de Wit E, Oude Vrielink JA, Elkon R, Melo SA, Leveille N, Kalluri R (2013). eRNAs are required for p53-dependent enhancer activity and gene transcription. Mol Cell.

[100] Lin L, Huang M, Shi X, Mayakonda A, Hu K, Jiang YY, Guo X, Chen L, Pang B, Doan N (2019). Super-enhancer-associated MEIS1 promotes transcriptional dysregulation in Ewing sarcoma in co-operation with EWS-FLI1. Nucleic Acids Res.

[101] Tsang FH, Law CT, Tang TC, Cheng CL, Chin DW, Tam WV, Wei L, Wong CC, Ng IO, Wong CM (2019). Aberrant super-enhancer landscape in human hepatocellular carcinoma. Hepatology.

[102] Cazet AS, Hui MN, Elsworth BL, Wu SZ, Roden D, Chan CL, Skhinas JN, Collot R, Yang J, Harvey K (2018). Targeting stromal remodeling and cancer stem cell plasticity overcomes chemoresistance in triple negative breast cancer. Nat Commun.

[103] You Z, Liu C, Wang C, Ling Z, Wang Y, Wang Y, Zhang M, Chen S, Xu B, Guan H, Chen M. LncRNA CCAT1 promotes prostate cancer cell proliferation by interacting with DDX5 and miR-28-5p. Mol Cancer Ther. 2019;18:2469–79. 10.1158/1535-7163.MCT-19-0095 31387890

[104] Wong RWJ, Ngoc PCT, Leong WZ, Yam AWY, Zhang T, Asamitsu K, Iida S, Okamoto T, Ueda R, Gray NS (2017). Enhancer profiling identifies critical cancer genes and characterizes cell identity in adult T-cell leukemia. Blood.

[105] Nasu Y, Benke A, Arakawa S, Yoshida GJ, Kawamura G, Manley S, Shimizu S, Ozawa T (2016). In situ characterization of Bak clusters responsible for cell death using single molecule localization microscopy. Sci Rep.

[106] Zhang W, Ge H, Jiang Y, Huang R, Wu Y, Wang D, Guo S, Li S, Wang Y, Jiang H, Cheng J (2020). Combinational therapeutic targeting of BRD4 and CDK7 synergistically induces anticancer effects in head and neck squamous cell carcinoma. Cancer Lett.

[107] Pei T, Huang X, Long Y, Duan C, Liu T, Li Y, Huang W (2019). Increased expression of YAP is associated with decreased cell autophagy in the eutopic endometrial stromal cells of endometriosis. Mol Cell Endocrinol.

[108] Wang LJ, Sun GZ, Chen YF (2019). LncRNA MSTO2P promotes proliferation and autophagy of lung cancer cells by up-regulating EZH2 expression. Eur Rev Med Pharmacol Sci.

[109] Qiao E, Chen D, Li Q, Feng W, Yu X, Zhang X, Xia L, Jin J, Yang H (2019). Long noncoding RNA TALNEC2 plays an oncogenic role in breast cancer by binding to EZH2 to target p57(KIP2) and involving in p-p38 MAPK and NF-kappaB pathways. J Cell Biochem.

[110] Das G, Shravage BV, Baehrecke EH. Regulation and function of autophagy during cell survival and cell death. Cold Spring Harb Perspect Biol. 2012;4. 10.1101/cshperspect.a008813 PMC3367545 22661635

[111] Yoshida GJ (2017). Therapeutic strategies of drug repositioning targeting autophagy to induce cancer cell death: from pathophysiology to treatment. J Hematol Oncol.

[112] Saitoh M (2018). Involvement of partial EMT in cancer progression. J Biochem.

[113] Tang B, Tang F, Wang Z, Qi G, Liang X, Li B, Yuan S, Liu J, Yu S, He S (2016). Overexpression of CTNND1 in hepatocellular carcinoma promotes carcinous characters through activation of Wnt/beta-catenin signaling. J Exp Clin Cancer Res.

[114] Yoshida GJ (2016). Emerging role of epithelial-mesenchymal transition in hepatic cancer. J Exp Clin Cancer Res.

[115] Hseu YC, Chang GR, Pan JY, Rajendran P, Mathew DC, Li ML, Liao JW, Chen WT, Yang HL (2019). Antrodia camphorata inhibits epithelial-to-mesenchymal transition by targeting multiple pathways in triple-negative breast cancers. J Cell Physiol.

[116] Viallard C, Larrivee B (2017). Tumor angiogenesis and vascular normalization: alternative therapeutic targets. Angiogenesis.

[117] Dai J, Wei R, Zhang P, Kong B (2019). Overexpression of microRNA-195-5p reduces cisplatin resistance and angiogenesis in ovarian cancer by inhibiting the PSAT1-dependent GSK3beta/beta-catenin signaling pathway. J Transl Med.

[118] Yuan Z, Bian Y, Ma X, Tang Z, Chen N, Shen M (2019). LncRNA H19 knockdown in human amniotic Mesenchymal stem cells suppresses angiogenesis by associating with EZH2 and activating Vasohibin-1. Stem Cells Dev.

[119] Spurlock CF, Shaginurova G, Tossberg JT, Hester JD, Chapman N, Guo Y, Crooke PS, Aune TM (2017). Profiles of Long noncoding RNAs in human naive and memory T cells. J Immunol.

[120] Wu M, Shen J (2019). From super-enhancer non-coding RNA to immune checkpoint: frameworks to functions. Front Oncol.

[121] Garcia-Diaz A, Shin DS, Moreno BH, Saco J, Escuin-Ordinas H, Rodriguez GA, Zaretsky JM, Sun L, Hugo W, Wang X (2017). Interferon receptor signaling pathways regulating PD-L1 and PD-L2 expression. Cell Rep.

[122] Zhu H, Bengsch F, Svoronos N, Rutkowski MR, Bitler BG, Allegrezza MJ, Yokoyama Y, Kossenkov AV, Bradner JE, Conejo-Garcia JR, Zhang R (2016). BET Bromodomain inhibition promotes anti-tumor immunity by suppressing PD-L1 expression. Cell Rep.

[123] Hogg SJ, Vervoort SJ, Deswal S, Ott CJ, Li J, Cluse LA, Beavis PA, Darcy PK, Martin BP, Spencer A (2017). BET-Bromodomain inhibitors engage the host immune system and regulate expression of the immune checkpoint ligand PD-L1. Cell Rep.

[124] Noriega-Guerra H, Freitas VM. Extracellular matrix influencing HGF/c-MET signaling pathway: impact on Cancer progression. Int J Mol Sci. 2018;19. 10.3390/ijms19113300 PMC6274944 30352967

[125] Zhou C, York SR, Chen JY, Pondick JV, Motola DL, Chung RT, Mullen AC (2016). Long noncoding RNAs expressed in human hepatic stellate cells form networks with extracellular matrix proteins. Genome Med.

[126] Ho LTY, Skiba N, Ullmer C, Rao PV (2018). Lysophosphatidic acid induces ECM production via activation of the Mechanosensitive YAP/TAZ transcriptional pathway in trabecular meshwork cells. Invest Ophthalmol Vis Sci.

[127] Yoshida GJ, Azuma A, Miura Y, Orimo A. Activated fibroblast program orchestrates tumor initiation and progression; molecular mechanisms and the associated therapeutic strategies. Int J Mol Sci. 2019;20. 10.3390/ijms20092256 PMC6539414 31067787

[128] Wang TH, Hsia SM, Shieh TM. Lysyl oxidase and the tumor microenvironment. Int J Mol Sci. 2016;18. 10.3390/ijms18010062 PMC5297697 28036074

[129] Levental KR, Yu H, Kass L, Lakins JN, Egeblad M, Erler JT, Fong SF, Csiszar K, Giaccia A, Weninger W (2009). Matrix crosslinking forces tumor progression by enhancing integrin signaling. Cell.

[130] Giussani M, Merlino G, Cappelletti V, Tagliabue E, Daidone MG (2015). Tumor-extracellular matrix interactions: identification of tools associated with breast cancer progression. Semin Cancer Biol.

[131] Hirata E, Girotti MR, Viros A, Hooper S, Spencer-Dene B, Matsuda M, Larkin J, Marais R, Sahai E (2015). Intravital imaging reveals how BRAF inhibition generates drug-tolerant microenvironments with high integrin beta1/FAK signaling. Cancer Cell.

[132] Yoshida GJ (2020). Applications of patient-derived tumor xenograft models and tumor organoids. J Hematol Oncol.

[133] Yoo KH, Hennighausen L, Shin HY (2019). Dissecting tissue-specific super-enhancers by integrating genome-wide analyses and CRISPR/Cas9 genome editing. J Mammary Gland Biol Neoplasia.

[134] Pefanis E, Wang J, Rothschild G, Lim J, Kazadi D, Sun J, Federation A, Chao J, Elliott O, Liu ZP (2015). RNA exosome-regulated long non-coding RNA transcription controls super-enhancer activity. Cell.

[135] Thandapani P (2019). Super-enhancers in cancer. Pharmacol Ther.

[136] Zhou H, Schmidt SC, Jiang S, Willox B, Bernhardt K, Liang J, Johannsen EC, Kharchenko P, Gewurz BE, Kieff E, Zhao B (2015). Epstein-Barr virus oncoprotein super-enhancers control B cell growth. Cell Host Microbe.

[137] Eliades P, Abraham BJ, Ji Z, Miller DM, Christensen CL, Kwiatkowski N, Kumar R, Njauw CN, Taylor M, Miao B (2018). High MITF expression is associated with super-enhancers and suppressed by CDK7 inhibition in melanoma. J Invest Dermatol.

[138] Pelish HE, Liau BB, Nitulescu II, Tangpeerachaikul A, Poss ZC, Da Silva DH, Caruso BT, Arefolov A, Fadeyi O, Christie AL (2015). Mediator kinase inhibition further activates super-enhancer-associated genes in AML. Nature.

[139] Geng M, Yang Y, Cao X, Dang L, Zhang T, Zhang L. Targeting CDK12-mediated transcription regulation in anaplastic thyroid carcinoma. Biochem Biophys Res Commun. 2019;520:544–5049. 10.1016/j.bbrc.2019.10.052 31615655

[140] Kennedy AL, Vallurupalli M, Chen L, Crompton B, Cowley G, Vazquez F, Weir BA, Tsherniak A, Parasuraman S, Kim S (2015). Functional, chemical genomic, and super-enhancer screening identify sensitivity to cyclin D1/CDK4 pathway inhibition in Ewing sarcoma. Oncotarget.

